# Policy and Fertility, a Case Study of the Quebec Parental Insurance Plan

**DOI:** 10.1007/s11113-024-09859-6

**Published:** 2024-05-05

**Authors:** Benoît Laplante

**Affiliations:** Centre Urbanisation Culture Société, Institut national de la recherche scientifique, Montréal, Québec Canada

**Keywords:** Parental leave, Fertility, Family policy, Difference in differences, Canada

## Abstract

In 2006, the Quebec government implemented a parental leave program more generous than the scheme available through the Canadian federal Employment Insurance (EI) program. It was aimed at maintaining the personal disposable income after a birth, especially for women whose income exceeds the maximum insurable earnings of EI. In this article, we assess whether the implementation of the Quebec Parental Insurance Plan (QPIP) was associated with an increase in the fertility in Quebec, especially for highly educated women. We use data from the rotating panels of the Canadian Labor Force Survey. We test the effect of the implementation of the QPIP on fertility by comparing Quebec and Ontario, which kept the federal EI scheme, before and after the implementation of the QPIP. We adapt the difference in differences method (DiD) to the modeling of the fertility schedule using Poisson regression. We estimate fertility by educational levels within each of the four groups of the DiD design by integrating the estimated fertility schedules. Our results show that the implementation of the QPIP was associated with an increase in fertility in Quebec. The magnitude of the increase varies by educational levels: 17% for women who did not complete secondary education, 46% for those who completed it, and 27% for women who earned a university diploma.

## Introduction

In Canada, paid maternity leaves were introduced in 1971 as part of the federal employment insurance program (EI). In 1997, the Quebec government initiated the implementation of a family policy whose design included the introduction of a genuine parental insurance plan. The Quebec plan was to be more generous than the EI maternity and parental leaves and, in a typically Canadian fashion, its implementation involved a conflict over what the Canadian constitution defines as the exclusive powers of the provincial legislatures. The government of Quebec won the argument and forced the federal government into signing an agreement by which the new Quebec Parental Insurance Plan (QPIP) would replace the provisions of the EI program for paid maternity and parental leaves in Quebec. Since 2006, parents living in Quebec are covered by the QPIP, while parents living elsewhere in Canada still receive their benefits from the federal EI.

The fact that the QPIP replaces the EI maternity and parental leaves in one Canadian province and not in the others creates a natural experiment that allows testing its effect using the difference in differences (DiD) method. Since giving birth is an event related to age and typically studied using age-specific rates, we adapt the DiD method to the modeling of the fertility schedule. Doing so permits us to estimate the total fertility rate within the groups defined for the estimation of the DiD equation. Given that one of the purposes of the QPIP is to reduce the opportunity costs of having children especially for highly educated women for whom these costs are higher, we further estimate the TFR by educational levels within each of the four groups defined for the DiD equation. This allows comparing the changes in the fertility of women according to their educational level before and after the implementation of the QPIP in Quebec and in a Canadian province where maternal and parental leaves are still provided by EI.

The article is organized as follows. First, we provide background information on family policy in Quebec, on the nature of the QPIP and the context in which it was developed and implemented, and on the relations between family policy and fertility. Second, we present our data and methods: the rotating panels of the Canadian Labor Force Survey (LFS) which provide the information needed to study births as events; the identification issues of our use of the implementation of the QPIP as a natural experiment; the need for an adaptation of the DiD approach that allows dealing with the rates that are at the core of demographic analysis; and the equation that implements this adaptation and its estimation. Then we present the results from our analyzes and those of some sensitivity checks. We end with a discussion.

## Background

### Family Policy in Quebec

After a failed attempt at overt pronatalism in the 1980s, the Quebec government published in 1997 a statement that initiated the implementation of a family policy that was to be based on three measures: the reform of financial support for families through the introduction of a unified child allowance, the development of early childhood education and care services, and the introduction of a new parental insurance plan. The choice of these measures was justified by the then still recent transformations of the family and of the labor market: 1. the increase in the labor force participation of women, especially mothers; 2. the increase in the proportion of single-parent families; and 3. increasing job insecurity and thus increasingly unstable sources of income for families through the proliferation of part-time employment, non-permanent employment, and self-employment. More generally, their analysis led the authors of the statement to make the reconciliation of work and family the major issue of family policy (Ministère du conseil exécutif, 1997).

By making gender equality and the reconciliation of work and family the main issues of its family policy and thus by choosing measures that favor women’s labor force participation and the sharing of duties within the couple, the Quebec government broke with the pronatalism that had inspired the universal birth allowance introduced in 1988 (Lapierre-Adamcyk, 2010; Mathieu, 2013). Quebec’s family policy was now taking at least part of its inspiration from the Nordic model, assuming that people want to have children and seeking to enable them to have the children they want in the contemporary economic and social context rather than inciting couples to have children (Roy, 2004; Roy & Bernier, 2006; Beaujot et al., 2013; Brauner-Otto, 2016; Mathieu et al., 2020).

The unified child allowance was introduced as early as 1997, but it has undergone several transformations since then, and the measures that replaced it—currently bundled under the name ‘Family allowance’—continue to co-exist with similar measures of the federal government, notably the Canada Child Benefit launched in 2006. From 1998 to 2018, the governments of both Quebec and Canada have increased their financial support towards families with children, both in a non-universalist fashion, the amount of support decreasing as market income increases. Since the end of the 1990s, low- and middle-income families living in Quebec have been better off than comparable families living elsewhere in Canada largely because Quebec’s financial support supplements the federal support available to all Canadian families (St-Cerny et al., 2018).

According to the 1997 policy statement, affordable childcare services were to be provided by subsidized independent community-led childcare centers which were to be non-profit organizations or cooperatives. Potential users of childcare services in any given area—a neighborhood, a town—were, and still are, encouraged to group together, create the required legal entity, write up a proposal for the Ministry in charge of childcare services based on the Ministry’s norms, find premises fulfilling the norms of the Ministry and within their budget, and hire specially trained early childhood educators and other staff assuming, of course, that the proposal is accepted and funded as the budget for childcare is limited. Under the current family policy—and unlike primary and secondary education—, childcare is not an entitlement, something the state must provide. For a reader familiar with the organization of childcare services and public preschool in some other country, say France, the burden placed on parents by the Quebec model is an obvious impairment to the development of childcare services. Not surprisingly, since the implementation of the model, the development of childcare services has been less than optimal and the slow growth of the community-led centers fostered the development of alternative providers, many private, some subsidized and other conventional profit-seeking corporations (Mathieu, 2019). Despite the original policy of promoting community-led centers, still in force, and the rise of alternative providers, the lack of affordable childcare is still an acute problem in Quebec. Comparing Ontario and Quebec, Lefebvre, Merrigan & Verstraete (2009) show that despite its limitations, the implementation of the Quebec childcare scheme increased women’s labor force participation. To our knowledge, no attempt has yet been made at assessing its impact on fertility and it is not the purpose of this article to do so.

### The Quebec Parental Insurance Plan

Canada is a federation, and the Canadian Constitution details the powers of the Federal Parliament on the one hand and of the provincial legislatures on the other hand. These provisions of the Constitution have been interpreted so that labor law is a provincial jurisdiction except for a small group of organizations: the federal government and its agencies, as well as banks, telecommunications, airlines, and a few other sectors of the economy which are deemed interprovincial by nature. In 1940, an amendment to the Constitution added unemployment insurance to the list of exclusive powers of the Federal Parliament. Thus, in Canada, the entitlement to the maternity and parental leaves is a matter of labor law and is regulated by the provincial labor law for most employees and the federal labor law for a few, whereas, apart from Quebec since the implementation of the QPIP, the maternity and parental leave benefits are regulated by the federal statute on ‘employment insurance’.

In 1971, the federal government introduced a 15-week maternity benefit as part of the unemployment insurance program. The duration of the maternity benefit was and still is different from the duration of unemployment benefits, but the maximum insurable earnings and the replacement rate were and have remained the same for maternity benefits and unemployment benefits. The details of these benefits changed over time, one of the most important changes being the introduction of parental benefits: 10 additional weeks of paid leave which could be shared between the mother and the father from 1991, and 35 from 2001. In 1991, unemployment insurance (UI) was renamed employment insurance (EI). Over the years, most Canadian provinces amended the provisions of their labor law so that employees under their jurisdiction may take full advantage of the maternity and parental benefits made available to them by the federal Employment Insurance Act. Ontario did so in by amending its Employment Standards Act several times: first in 1972 to introduce a 17-week maternity leave, then in 2000 to introduce a 35- to 37-week parental leave, and finally in 2017 to match the most recent changes in the duration of the EI parental benefit. If Ontario hadn’t amended its labor law to match the changes in the EI maternity and parental benefits, it would not have been possible to use it to compare the QPIP with the federal EI scheme. Table 4, in the Appendix, provides an overview of the evolution of the maternal and parental benefits of employment insurance from 1991 onwards.

The QPIP is the result of a struggle initiated in 1988 and led by the *Regroupement pour un régime québécois d’assurance parentale* (Coalition for a Quebec Parental Insurance Plan), a coalition made up of 16 organizations, mainly family-related community organizations, women’s groups, and unions. The Quebec government began to show support for the QPIP in 1996 and made it an element of its new family policy in 1997. At that time, maternal and parental leave benefits existed within the federal employment insurance program. The benefits of the QPIP were planned to be more generous than those of EI and the program was to be funded with premiums paid by employers and employees as EI was. Thus, the government of Quebec initiated discussion with the federal government with the intent of convincing the federal government to transfer to the QPIP the proportion of the EI premiums collected in Quebec which were used to pay for the EI maternal and parental benefits in Quebec. The federal government did not agree but had to change its mind after the Quebec Court of Appeal ruled that according to the Constitution, providing such a benefit was among the powers granted to the provincial legislatures (Conseil de la famille et de l’enfance, 2008; Giroux, 2008, 2016; Beauchemin, 2016). The Quebec Parental Insurance Plan (QPIP) came into effect in January 2006 and was thus the last of the three measures to be implemented.

The main motivation of the QPIP is to help women being mothers and workers at the same time by allowing them to remain in the labor force while they take care of a newborn or an infant. It relies on the assumption that women will be back to their job at the end of the leave. It is a mandatory insurance plan funded by contributions from employers and employees. Labor law has been harmonized with the QPIP. The right to a maternal or parental leave is a matter of labor law and is distinct from the entitlement to the benefits, but the two are coordinated so that an employer cannot lay off a woman who takes a maternity or a parental leave and receive the benefits from the plan.

The QPIP tries to achieve its goal by maintaining the personal disposable income after contributions and taxes of a large proportion of working women, including highly educated women who do well on the labor market and whose income exceeds the maximum insurable earnings of EI (Ministère du conseil exécutif, 1997, p. 27). From a theoretical perspective, the QPIP relies on the now common view that the indirect cost of a child for a woman increases with her human capital while upending Becker’s (1985) practical conclusions. In other words, the QPIP rests on the idea that women and especially highly educated women refrain from having a child they want because the loss of income is too large under EI. Setting a replacement rate higher than that of EI increases the benefits for all but setting the maximum insurable earnings of the QPIP higher than those of EI increases the benefits of those, primarily highly educated women in this context, who earn more than the EI maximum insurable earnings.

Since its beginning, the provisions of the QPIP have consistently been more generous than those of EI. The maximum yearly insurable earnings were 57,000 CAD in the first year of the plan while they were 39,000 CAD for EI; in 2022, they stood at 88,000 CAD for the QPIP while they were 60,300 CAD for EI. The maximum insurable earnings and replacement rate of the QPIP are comparatively high. For instance, in 2021, the average annual income for university graduates excluding managers was 83,678 CAD in the non-unionized private sector and 78,813 CAD in the unionized one (Institut de la statistique du Québec, 2022a, 2022b), while the maximum insurable earnings of the QPIP that year was 83,500 CAD. Thus, in 2022, a woman who had earned the maximum insurable earnings or more received 52,885 CAD for a 50-week leave from the QPIP but 33,165 CAD from EI.

The QPIP has other features. It explicitly promotes the sharing of childcare between women and men. From the outset, it allowed parents to divide the leave between them, while strictly reserving a fraction of it for the father who cannot transfer it to the mother. These five weeks of paternal leave are meant to ‘seduce’ fathers into the care of their young children and to signal employers that parental responsibilities and parental leaves are for their male staff too.

Finally, the QPIP offers options that allow trading off between the duration and the replacement rate. The basic option comprises 18 weeks for the mother and 5 weeks for the father with a 70% rate, plus 7 weeks at 70% and 25 weeks at 55% that can be used either by the mother or the father; see Table 5 in the Appendix for an overview of the QPIP.

### Family Policy and Fertility

**Fig. 1:**
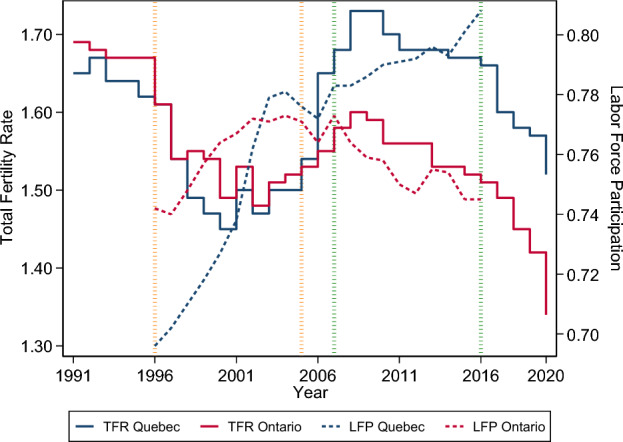
Total fertility rate (TFR), Quebec and Ontario, 1991–2020, left axis. Women aged 15 to 44 labor force participation rate (LFP), Quebec and Ontario, 1996–2016, right axis. The dotted orange vertical lines delimit the period before the implementation of the QPIP used in the DiD equations; the dotted green ones the period after. *Source:* Adapted from Chart 5 in Moyser and Milan (2018). Data on the labor force participation rate from Moyser and Milan (2018); the rates were computed for this publication as Statistics Canada does not routinely publish this rate for this age group. Data on the total fertility rate from Statistics Canada. Table 13-10-0418–01 Crude birth rate, age-specific fertility rates and total fertility rate (live births). DOI: https://doi.org/10.25318/1310041801-eng

The current Quebec family policy is aimed at supporting the reconciliation of family and work primarily through the support it gives to working mothers (Roy & Bernier, 2006). Until now, its success has been measured mainly by the increase in the labor force participation of women which grew faster in Quebec than in neighboring Ontario after the implementation of low-cost childcare (Lefebvre et al.,^2009^). A descriptive study focusing on the evolution of fertility and labor force participation in Quebec and Ontario since the 1990s suggests that the Quebec family policy is the likely cause of their marked increase in Quebec over that period and the persistence of the difference in fertility between the two provinces especially after 2008 (Moyser & Milan, 2018). Figure 1 is adapted from this study. From it, one can see that in 1996, fertility as measured by the total fertility rate (TFR) was similar in Quebec and Ontario—1.61—, but female labor force participation was higher in Ontario than in Quebec—respectively 74.2% and 69.6%, thus 6.6% higher in Ontario. From 1996 to 2000, fertility declined in both provinces, but more rapidly in Quebec—down to 1.49 and 1.46 respectively, thus by 7.4% and 9.3% —, while female labor force participation increased, but more rapidly in Quebec—from 69.6% to 72.6% in Quebec and from 74.2% to 76.4% in Ontario, thus by 4.3% and 2.9%. Women’s labor force participation in Quebec surpassed that of Ontario around 2003—77.9% and 77.1% respectively—and remained slightly higher until 2007. Fertility increased with fluctuations and in a similar manner in both provinces from 2001 to 2008, but from 2006 onwards, it is higher in Quebec—1.65 in Quebec in 2006 and 1.55 in Ontario. Female labor force participation peaked in 2007 in Ontario at 77.3% and then declined with fluctuations, while it continued to increase slowly in Quebec where it became noticeably higher—80.8% in Quebec and 74.5% in Ontario in 2016. Fertility has been declining in both provinces since 2009, but the gap that existed in 2008 remains and might be increasing: from 2008 to 2018, fertility was about 8% higher in Quebec than in Ontario but the difference reached 10.6% in 2020 and 13.4% in 2020. In Quebec, it is as if family policy measures had clearly achieved their objective—to allow women to be mothers and to have a job—between 1996 and 2008, but in a more qualified way afterwards. Since 2009, in Quebec, women’s labor force participation has increased, but fertility has declined, while in Ontario, both have declined.

## Data and Methods

Measuring the effect of a single family policy measure on fertility is not an easy task. It is generally accepted among scholars that the difference in fertility between developed countries that support working mothers, promote gender equality, and facilitate reconciliation between family and work, and those that don't do it is explained by the existence and effectiveness of these measures taken as a whole. Research supporting this interpretation is based on comparisons between countries (Harknett et al., 2014; Fernández Soto et al., 2020; Wesolowski, 2020; Bein et al., 2021) or between jurisdictions within a country (Hook & Paek, 2021).

The impact of a new measure can be assessed through using methods developed to take advantage of natural experiments (Bassford & Fisher, 2020; Lyssiotou, 2021; Milligan, 2005). However, this approach is not well suited to studying the effects of policies whose details change over time, and for this reason it is not widely used in societies where measures have been in place for some time. In such cases, more credibility is given to studies that rely on individual longitudinal data that link events such as births to the specific provisions of the measures and the use made of them by individuals and couples (Neyer & Andersson, 2008; Comolli et al., 2021). This type of study was possible in Canada when Statistics Canada was maintaining household panel surveys: the data from such studies has been used to study the effect of family policy measures on women’s labor force participation (Lefebvre et al., 2009), on child development (Haeck et al., 2015), and on fertility (Laplante et al., 2010, 2015).

The implementation of the QPIP in 2006 offers a not so common opportunity to assess the effect on fertility of a family policy measure using an approach that takes advantage of a natural experiment. The QPIP was implemented at a single moment, its benefits are markedly different from those of EI, it is universal in the sense that it is it is a true entitlement—the number of women who can use is not constrained by a budget limit or a cap of the number of recipients—and that women whose market income exceeds the amount of the maximum insurable earnings are not excluded from it, and, finally, it replaced EI for people living in Quebec but not elsewhere in Canada. So far, this opportunity has been used to assess its effect on the uptake of parental leave by fathers—the QPIP increases it—(Margolis et al., 2019a, 2019b), fathers’ participation—it increases it—(Patnaik, 2019), and on union dissolution—it decreases it—(Margolis et al., 2021) but not on fertility.

The main argument that could be raised against studying the implementation of the QPIP as a natural experiment is that it was part of a three-pronged family policy and was thus one element of an environment policy resulting from its combination with the two other measures. The rejoinder is empirical: there is no evidence that fertility started to evolve differently in Quebec than in Ontario before the implementation of the QPIP even though Quebec had implemented the first two measures of its family policy at the end of the 1990s. One could still argue that the increase in Quebec fertility that starts in the year of the implementation of the QPIP is not an effect of the QPIP per se but an effect of the addition of the QPIP to the first two measures. This argument cannot be rejected easily. One can simply reply that the scope of the financial support and of the reduced-cost childcare services are more limited than that of the QPIP, the first one because it is focused on low-income families and the second, because by the very design of the Quebec model, the provision of childcare cannot meet demand.

### Identification Issues

*Policy endogeneity.* As explained above, the QPIP was included among the three measures of the 1997 policy statement after almost ten years of advocacy from women’s groups. Because of the complexity and length of the dealings with the federal government, it was the last of the three to be implemented, almost ten years afterwards. Thus, two relevant changes occurred in Quebec between 1997 and 2006, the implementation of the first two measures of the 1997 statement: the unified child allowance in 1997, and the development of early childhood education and care services which was initiated as early as 1997 but is still not completed in 2023.

Thus, the QPIP had been advocated for about 18 years when it was implemented, and about half of this delay was caused by the dealings between the federal and Quebec governments, which can be reasonably assumed as exogenous to fertility. Furthermore, as can be seen form Fig. 1, until the implementation of the QPIP, Quebec and Ontario fertility were following the same trend despite the implementation of the first two measures of the 1997 statement. One might suggest that the somewhat higher fertility in Quebec between 1997 and 2002 might be related to the implementation of the first two measures, but if it were so, the effect would have been modest and short lived. Previous studies have shown that affordable childcare services have increased the labor force participation of Quebec women (Lefebvre et al., 2009), which can also be seen in Fig. 1, but none has attempted to estimate of the effect of the unified child allowance or of the affordable childcare services on fertility and, again, Fig. 1 shows that such attempts would most likely be dead ends.

As explained above, changes in the duration of Ontario maternity and parental leaves occurred in 1972, 2000 and 2017 to match the changes in the duration of the corresponding EI benefits. The only change relevant for our study occurred in 2000; this was before the implementation of the QPIP and Quebec changed its labor law to match the changes in EI benefits as Ontario did. The 2000 changes in the duration of EI benefits and the according changes in the duration of the unpaid leaves might explain the increase in fertility in the two provinces from 2002 to 2009. It cannot explain the ‘jump’ in Quebec fertility that occurred in 2006 and resisted the decreasing trend that started in both provinces in 2009.

Hence, there is no plausible influence of changes in policy measures that might interfere with the effect of the implementation of the QPIP.

*Common trends assumption.* Figure 5 in the Appendix displays the total fertility rate, its logarithm and its exponential in Quebec and Ontario before and after the implementation of the QPIP. The trends are parallel in all three scales. Figure 6 in the Appendix displays the difference between the total fertility rates of Quebec and Ontario from 1991 to 2020. The difference increases in 2006, the year of the implementation of the QPIP.

The total fertility rates plotted in Fig. 1 and the upper part of Figure 5 come from the vital statistics computed and published by Statistics Canada. To check the common trends assumption by educational groups, we computed the TFR by educational levels for the two provinces and the two periods used in the analysis from the LFS data. This pushes the LFS samples to their limit. The result can be seen in Figure 7 in the Appendix. Figure 8 in Appendix makes the patterns easier to see. In all educational levels, the average TFR is lower in Quebec than in Ontario prior to the implementation of the QPIP. The average Quebec TFR is higher after the implementation of the QPIP than before in all educational levels. After the implementation of the QPIP, the average Quebec TFR is higher than that of Ontario among secondary and university-educated women. It is lower than that of Ontario among women who did not complete secondary education and those who have a non-university postsecondary diploma, but closer to it than before the implementation of the QPIP. The yearly difference between the provincial TFRs is noisy but the mean differences show an increase, although small in some cases, within each educational level. All of this is consistent with the overall result of the analysis and consistent with the finding that the effect of the QPIP varies across educational levels.

### Data

As in previously published studies on related topics, we compare Quebec to Ontario. Ontario and Quebec are the two largest Canadian provinces by the size of their population; they share a border and unlike some other Canadian provinces, they both have a diversified economy. There are noticeable differences between the two, the most salient being that Quebec is the only Canadian province where most people speak French rather than English. Nonetheless, given the similarities between the two, it makes more sense to compare Quebec with Ontario alone than with all the other provinces as these, because of their particularities, would make a more heterogenous control group than Ontario alone.

We focus on women aged between 20 and 49 who are employed and are living in a heterosexual cohabiting relationship, married or not, where the partner or spouse has some employment income, and are at risk of having a child when they enter an LFS panel. For these women, our dependent variable is the hazard of giving birth while they are still in the panel.

We use the Quebec and Ontario subsamples of the detailed microdata from the Labor Force Survey (LFS). The LFS is the official source of monthly estimates of total employment and unemployment in Canada. Apart from the Census, the LFS is the only mandatory household survey in Canada. This explains why, despite its focus on employment, it is also one of the main sources of information on the socio-demographic characteristics of the working-age population, namely age, education, marital status, and family situation. The survey uses rotating panels: selected individuals remain in the sample for six months (Statistics Canada, 2017). The detailed LFS microdata are grouped into monthly files. People living in the same dwelling are grouped into families; identifiers allow following individuals from month to month. One variable indicates the month in which a person was included in the sample for the first time. We use this variable to locate the moment at which a woman is first observed at risk of having a child and to locate births.

The sample we use for the DiD analysis is made of 187,023 women of which 4572 gave birth while in a rotating panel of the LFS and after the first month they were in it. The sample comprises 34,880 women from Quebec and 58,704 from Ontario before the implementation of the QPIP, and 36,601 from Quebec and 56,838 from Ontario in the period following its implementation. The left portion of Table 6 provides a description of the four groups on a few relevant characteristics.

### Model

Difference in differences is a quasi-experimental method widely used in program evaluation to assess the effect of a policy. It uses a ‘natural experiment’ that allows comparing two populations before and after the implementation of the policy—known as the ‘treatment’—in one of the populations. It was introduced by Card and Krueger (1994) in a study of the effect of a wage increase on the employment level in the fast-food industry. It is typically used with a continuous dependent variable—full-time equivalent workers per store in the original article—and four groups formed by crossing the two populations and the two periods—New Jersey and Pennsylvania before and after an increase in the minimum wage in New Jersey. The core of the method consists in estimating whether the change in the value of the dependent variable in the two populations between the two periods is the same or not. This is done by estimating a multiple regression equation that includes three terms: one binary variable for the two populations, one for the two periods and one for the product of the first two terms, known as the ‘difference in differences coefficient’. Estimating the equation without the third term assumes that the change in the dependent variable is the same in the two populations. Estimating the equation with the third allows testing this assumption: if the third term is statistically different from zero, the change is larger in one population, hopefully the one in which the policy was implemented. Thus, DiD is essentially an application of the technique developed for testing the assumption of additivity in the two-way analysis of variance (Brandt, 1933; Yates, 1934). For this reason, the DiD method can be used with most if not all linear models.

Birth is an event and demography studies the occurrence of events by envisioning them as processes governed by rates: fertility rates, mortality rates, migration rates for the core demographic events, nuptiality rates, divorce rates and so on for the more specific events studied by family demography. The most common measure of fertility is the total fertility rate which is the sum of age-specific fertility rates over the span of reproductive years. Poisson regression allows modeling the rates that govern occurrence of events such as births, computing predicted age-specific rates and predicted TFR, and interpreting the results within the conceptual framework of demography. Thus, from the perspective of demography, using Poisson regression when studying fertility is conceptually coherent and allows dealing with the core question while using a form of regression that models probability, as in the original formulation of the DiD model, is not conceptually coherent with the discipline and does not deal with the core question. While some approaches to the statistical modeling of natural experiments have been developed using non-linear models, such as the adaptation of regression discontinuity to epidemiological analysis using survival models by Bor et al. (2014), we found no example of the DiD approach based on Poisson regression or on any other linear model that could be utilized to study fertility within the conceptual framework of demographic analysis. We propose here an application of it to the study of fertility using Poisson regression.

Specifically, we aim at assessing whether the implementation of the QPIP influenced fertility in Quebec. Given that EI rules changed over time—notably in 2006, the very year the QPIP was implemented—and then in 2018 and 2019, the best choice is to define the period before the ‘treatment’ as ranging from 1996 to 2005, and to define the period after the ‘treatment’ as ranging from 2007 to 2017 (See Table 4 for more details on the two periods).

Using DiD and assuming a Poisson statistical distribution, the hazard of giving birth can be modeled as

1 $${\text{ln}}\left(\lambda \right)={{\beta }_{0}+\beta }_{Y}Y+{\beta }_{T}T+{\beta }_{YT}YT,$$

where ln(*λ*) is the natural logarithm of the hazard of giving birth, *Y* (from ‘year’) stands for the period—before or after the implementation of the QPIP—, *T* (from ‘treatment’) stands for the place—Ontario or Quebec, and *β*_*YT*_ is the difference in differences coefficient. In this equation, given that we restrict the sample to women in their reproductive years, the dependent variable—ln(*λ*)—is the logarithm of the general fertility rate.

The DiD approach can be extended to design a more realistic modeling of fertility. The first step of such an extension is replacing the origin of the equation with a baseline smoothed fertility schedule. This turns Eq. 1 into a statistical model based on a decrement table where the fertility schedule is akin to the baseline hazard of a survival model. Using a quadratic function of the age of the woman, this leads to

2 $${\text{ln}}\left(\lambda \right)=\left[{\alpha }_{{0}}+{\alpha }_{{1}}A+{\alpha }_{{2}}{A}^{2}\right]+\left[{\beta }_{Y}Y+{\beta }_{T}T+{\beta }_{YT}YT\right]+\sum_{j=1}^{n}{\gamma }_{j}{X}_{j},$$

where *A* is the age of the woman and *X*_*j*_ represents additional independent variables (‘controls’) *if any*. In this equation, the dependent variable—ln(*λ*)—is now the logarithm of the age-specific fertility rate. The schedule is assumed to have the same shape in the four groups and differs only by its vertical location which is determined by the values of the three coefficients associated with the conditional relation that implements the DiD. Later in this section we will see that we estimate Eq. 2, as well as Eqs. 3, 4 and 4s using sampling weights and control variables, but also using propensity score weights without control variables.

Equation 2 is an improvement from Eq. 1, but a limited one as it is known that in contemporary societies, the population of women in their reproductive age is not homogenous and that the fertility schedule varies primarily across educational levels—women with more education have their children at a later age—and that such variation leads to differences in horizontal location that cannot be modeled using a linear effect. This can be accommodated using a series of four baseline smoothed fertility schedules, one for each educational level:

3 $${\text{ln}}\left(\lambda \right)=\sum_{i=1}^{4}\left[{\alpha }_{{0}_{i}}{E}_{i}+{\alpha }_{{1}_{i}}{E}_{i}A+{\alpha }_{{2}_{i}}{E}_{i}{A}^{2}\right]+\left[{\beta }_{Y}Y+{\beta }_{T}T+{\beta }_{YT}YT\right]+\sum_{j=1}^{n}{\gamma }_{j}{X}_{j}.$$

Here *E* represents the educational level, and *A*, the age of the woman. The dependent variable is still the age-specific rate. This equation is akin to a stratified hazard model based on the Poisson distribution in which the effects of the independent variables are assumed to be the same in all strata which, in our case, means that the fertility schedule varies first according to the educational level and that other differences in the actual fertility schedule should be accounted for by differences in time, in places, in the parental leave plan or in some other independent variable.

The approach can be further extended by allowing the DiD terms to take different values for each of the four groups defined by place and period. Formally, the sum of the baseline and DiD terms in Eq. 3 is replaced with their product:

4 $${\text{ln}}\left(\lambda \right)=\sum_{i=1}^{4}\left[{\alpha }_{{0}_{i}}{E}_{i}+{\alpha }_{{1}_{i}}{E}_{i}A+{\alpha }_{{2}_{i}}{E}_{i}{A}^{2}\right]\times \left[{\beta }_{Y}Y+{\beta }_{T}T+{\beta }_{YT}YT\right]+\sum_{j=1}^{n}{\gamma }_{j}{X}_{j.}$$

This equation keeps the structure of the comparison between the groups, but as the terms that implement it are not constrained to be the same for the four groups, it is no longer a test of additivity and, thus, no longer a DiD model. However, by keeping the comparison structure of the DiD method while using a more flexible approach to the estimation of the conditional fertility schedules, it allows estimating them in a more realistic fashion. Thus, once the effect of the policy has been assessed using Eq. 3, Eq. 4 can be used to estimate the conditional fertility schedules and, more importantly, to estimate the corresponding conditional TFR by integration. Finally, replacing the quadratic specification of the fertility schedules in Eq. 4 with cubic splines allows still more realistic estimation of the conditional fertility schedules and TFR. We will refer to the cubic spline specification as Eq. 4s.

The DiD approach relies on the assumption that the value of the dependent variable varies over time in a parallel fashion in the two populations before and after the implementation of the new policy. In the ideal case, the value of the dependent variable would be stable in the two populations ‘before’ and ‘after’, the only change being an increase, or a decrease, soon after the change in policy. Figure 1 shows that our case is not ideal but is close enough to fulfill the assumption. In the period ‘before’ the implementation of the QPIP, the TFR was higher in Ontario than in Quebec most of the time, the magnitude of the gap changing over time. During this period, the TFR varied in a convex fashion in both provinces, reaching its lowest value—1.45—at the end of the 2000 in Quebec and two years later in Ontario—1.48. The TFR started to rise slightly in both provinces after 2002, its value remaining higher in Ontario. The TFR rose dramatically in Quebec in 2006, the year of the implementation of the QPIP, and again in 2007. The TFR increased in Ontario too in 2006 and 2007, but in a much lesser way. It reached its highest value in 2008 in both provinces, clearly higher in Quebec—1.73—than in Ontario—1.60. The TFR started to decrease in both provinces after 2008, but the gap between the two remained the same.

Our first objective is to assess the overall effect of the QPIP on the fertility of working women; our second one is to evaluate the extent to which this effect varies across educational levels. Given that we estimate the fertility schedule by educational levels within each of the four groups of the DiD design and that we want to estimate the average conditional TFR within educational levels, we have no use for additional independent variables. However, our dependent variable may be related with other potential independent variables, and it would be cautionary to estimate the DiD coefficient while taking such potential relations into account. Stuart et al. (2014) provides an alternative way of doing so. The strategy is akin to what is known as standardization in demography: it uses propensity score weighting to remove the effect of compositional differences between the DiD groups that may influence the dependent variable or, as the authors write it, “to control for confounding due to observed covariates that differ either across groups in the 'before' period, or even over time due to changes in group composition” (Stuart et al., 2014, p. 81). The use of the technique is straightforward. First, estimate a multinomial logistic regression in which the dependent variable is made of the four groups of the DiD design using whatever independent variables which might have an impact on the dependent variable of the DiD coefficient. Second, compute the propensity weight for each case as the quotient of the predicted probability of belonging to the standard group—here, Quebec before the implementation of the QPIP –, and of its predicted probability of belonging to the group it belongs to. Third, estimate the DiD equation using these weights. The DiD coefficient resulting from this estimation should be free of compositional effect. Comparing the distribution or means of the independent variables in the unweighted sample or, as in our case, with the sample weighted using the sampling weights, should show that the four groups are close to isomorphic for the independent variables. This can be seen in Table 6 which provides a description of the four groups of the DiD design first using sampling weights and second using the propensity weights.

As its name suggests, the LFS focuses on employment. It collects very detailed information on employment and some information on its causes and consequences, such as the level of education and the “usual [weekly] wages or salary of employees at their main job”. Except for these and the detailed composition of the household, which is essential for the weighting—and, for us, allows observing births and gives some information on previous births—, it contains little information that is relevant for the study of fertility. When estimating Eq. 2 using sampling weights, we use the woman’s and the man’s educational level, the number of children of each age group living in the household, and the income of each spouse or partner in 2020 inflation-adjusted Canadian dollars as control variables; when estimating Eqs. 3, 4 and 4s, the woman’s educational level is not used as a control variable as it is used to model the fertility schedule by educational level of the woman. The propensity score weights are estimated using the same sets of control variables in the multinomial logistic regressions. Among the characteristics that would have been of interest in our analysis but are not available in the data we use are the legal marital status —which was not collected before November 1999 —, immigration status —which was not collected before 2006 — and income from sources other than employment.

## Results

**Fig. 2:**
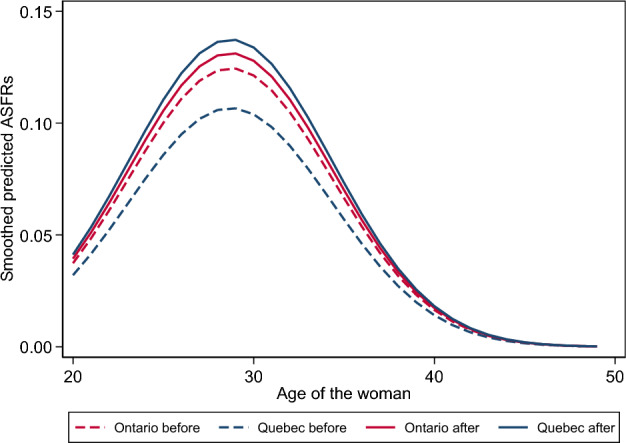
Age-specific fertility rates from Eq. 2, Quebec and Ontario, before and after the implementation of the QPIP

Table 1 reports the value of the difference in differences coefficient *β*_*YT*_ in Eqs. 2 and 3 expressed in multiplicative form. Its magnitude is the same in both equations and whether the estimation uses sampling weights or propensity weights—about 1.2—and is statistically different from 0. Thus, the implementation of the QPIP is truly associated with the increase in fertility in Quebec. Figure 2 shows that the Quebec fertility schedule predicted from the estimates of Eq. 2, lower than that of Ontario before the implementation of the QPIP, became indistinguishable from it afterwards. According to Eq. 2, the (conditional) TFR of partnered women aged between 20 and 49 who were in the labor force would have been the same in Ontario and Quebec after the implementation of the new plan.

**Table 1:** Value of the difference in differences coefficient

	Estimation with sampling weights and control variables		Estimation with propensity weights^a^	
	Equation 2	Equation 3	Equation 2	Equation 3
${e}^{{\beta }_{YT}}$	1.221**	1.234**	1.235***	1.222**

^a^The propensity weights are estimated within period, provinces and educational levels of the woman using multinomial logistic regression with the man’s educational level, the number of children of each age group living in the household and the income of each spouse or partner in 2020 inflation-adjusted Canadian dollars as independent variables. The equations estimated with sampling weights use the same set of characteristics as control variables. The coefficients associated with the control variables are reported in Table 7

*p < 0.05; **p < 0.01; ***p < 0.001

**Fig. 3:**
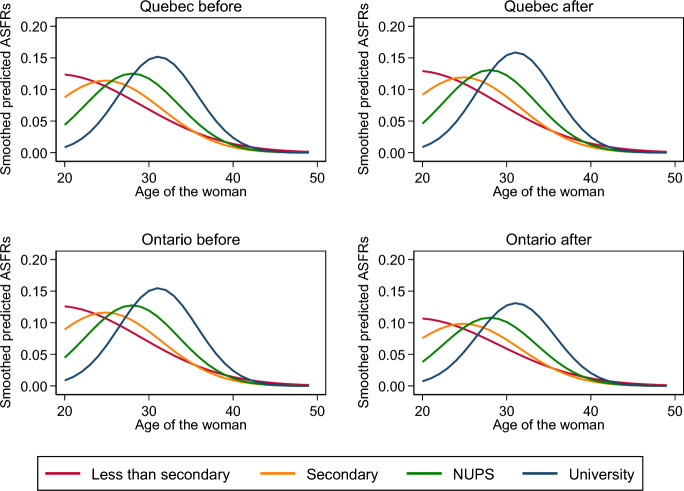
Age-specific fertility rates by educational level of the woman from Eq. 3, Quebec and Ontario, before and after the implementation of the QPIP

**Fig. 4:**
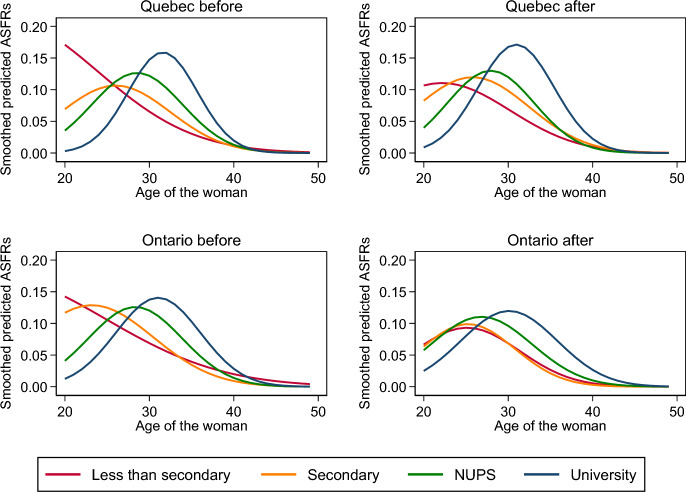
Age-specific fertility rates by educational level of the woman from Eq. 4, Quebec and Ontario, before and after the implementation of the QPIP

**Fig. 5:**
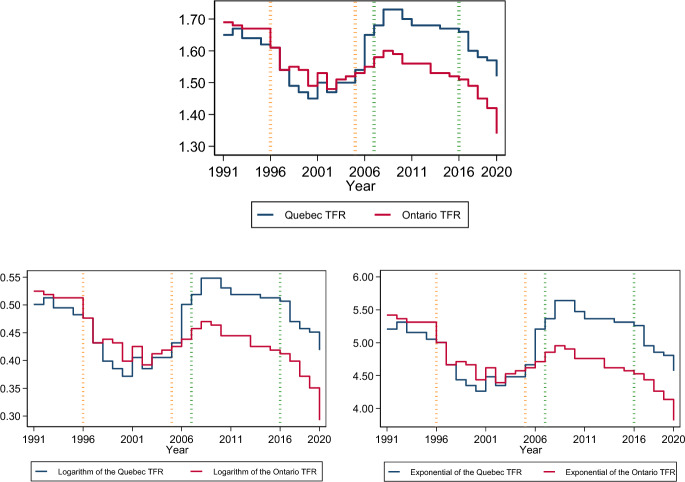
Total fertility rate, logarithm of the total fertility rate and exponential of the total fertility rate, Quebec and Ontario, 1991–2020. Source: Statistics Canada. Table 13–10-0418–01, Crude birth rate, age-specific fertility rates and total fertility rate (live births), DOI: https://doi.org/10.25318/1310041801-eng

**Fig. 6:**
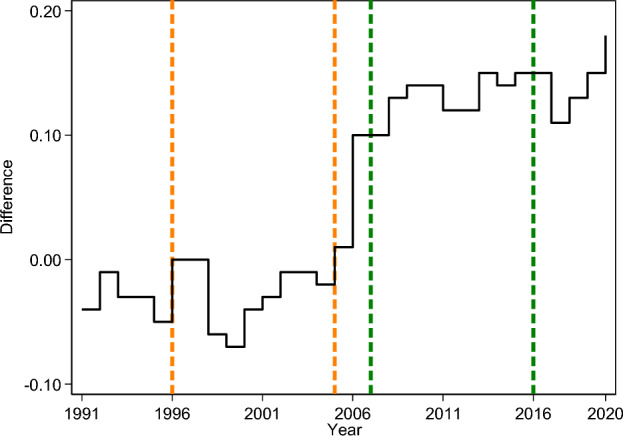
Difference between the total fertility rates of Quebec and Ontario, 1991–2020. Source: Statistics Canada. Table 13–10-0418–01, Crude birth rate, age-specific fertility rates and total fertility rate (live births), DOI: https://doi.org/10.25318/1310041801-eng

**Fig. 7:**
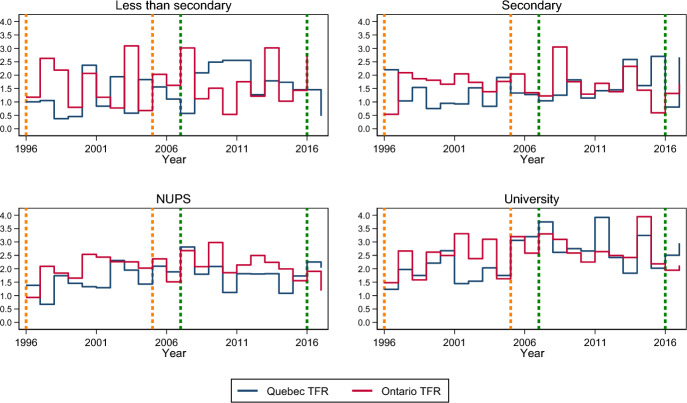
Total fertility rate by province, year, and educational levels estimated using the data from the Canadian Labor Force Survey. Quebec and Ontario, 1996–2017

**Fig. 8:**
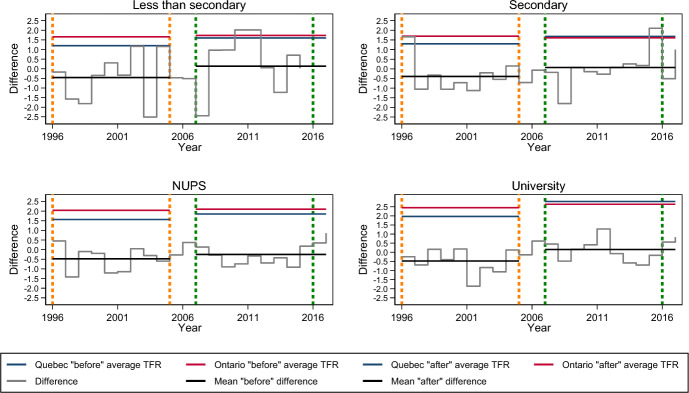
Average total fertility rate by province, period, and educational levels, yearly difference between the provincial total fertility rates by educational levels and mean within periods differences. Quebec and Ontario, 1996–2017. Estimated using the data from the Canadian Labor Force Survey. Quebec and Ontario, 1996–2017

Figure 3 displays the fertility schedule by educational levels in Quebec and Ontario before and after the implementation of the QPIP as estimated using Eq. 3. Equation 3 constrains the logarithm of the fertility schedule—the ASFRs—to differ between periods and provinces in an additive fashion: it imposes them the same shape and the same horizontal location. Given the equation, their shape differs little, the main differences being in their vertical location. Thus, the graph makes clear that assuming the same horizontal location for the whole population is ill-advised as the maximum of the curve moves to the right as the educational level increases. Figure 4 displays the fertility schedule by educational levels estimated from Eq. 4. The shape and the location of the curves vary across groups, showing that Eq. 3 oversimplifies the effects of the new plan. Using Eq. 3 to assess that the implementation of the QPIP had an effect makes sense, but not to assess the magnitude of this effect.

Table 2 reports the conditional TFR by educational levels in Quebec and in Ontario before and after the implementation of the QPIP estimated by integrating the fertility schedules from Eqs. 3, 4 and 4 s. According to the estimates of Eq. 3, the conditional TFR varies by educational levels: fertility is the highest among the women who hold a university diploma, lower among those who hold a non-university postsecondary diploma, still lower among those who completed secondary education and the lowest among those who did not complete secondary education, although the difference between the last two groups is close to zero. By design, the pattern is the same in Quebec and in Ontario, before and after the implementation of the new plan; the reversal of the order between the two groups of low-educated women in Ontario before the implementation of the QPIP is a consequence of the small difference between the two groups. In Quebec, the conditional TFRs are higher after the implementation of the new plan than before, while they are slightly smaller in Ontario. The conditional TFRs are higher in Ontario than in Quebec before the implementation of the QPIP but are higher in Quebec afterwards.

**Table 2:** Conditional total fertility rates by educational levels and provinces before and after the implementation of the QPIP from Eqs. 3, 4 and 4s

	Equation 3				Equation 4				Equation 4s			
	Quebec		Ontario		Quebec		Ontario		Quebec		Ontario	
	B	A	B	A	B	A	B	A	B	A	B	A
LTS	1.24	1.50	1.46	1.44	1.16	1.35	1.56	1.55	1.15	1.34	1.54	1.53
Secondary	1.26	1.53	1.49	1.46	1.14	1.62	1.57	1.42	1.14	1.66	1.57	1.43
NUPS	1.39	1.68	1.64	1.61	1.45	1.58	1.64	1.61	1.45	1.57	1.63	1.63
University	1.50	1.82	1.78	1.74	1.65	1.91	1.74	1.63	1.54	1.95	1.72	1.63

The conditional total fertility rate is computed by integration of the corresponding estimated fertility schedule. *LTS* Less than secondary, *NUPS* Non-university postsecondary education, *B* Before the implementation of the QPIP, *A* After the implementation of the QPIP

The conditional TFRs based on the estimates of Eq. 4 tell a somewhat different story. Because of the flexibility of cubic splines, the conditional TFRs computed from the estimates of Eq. 4s should be the most accurate. They are not very different from those computed from the estimates of Eq. 4 and they lead to similar conclusions. The differences between educational levels are greater in Quebec than in Ontario before and after the implementation of the QPIP, although they are smaller after. In Ontario, fertility has declined or remained the same before and after the implementation of the QPIP among women of all educational levels whereas it has increased among women of all educational levels in Quebec.

Table 3 synthesizes the effect of the QPIP by educational levels and provinces in a way that emphasizes the differences between the constraints imposed by the equations. The effect is the same for all educational levels in Eq. 3 but higher in Quebec than in Ontario; it varies across educational levels in Eqs. 4 and 4s. Given that there are no constraints on the variation of the effect across educational levels, little constraints on the shape of the fertility schedule and none on its horizontal location, Eq. 4s provides the most accurate estimation of the magnitude of the effect of the implementation of the QPIP: it increased fertility by 17% among women who did not complete secondary education, by 46% among women who completed it, by 8% among women with non-university postsecondary education, and by 27% among women who earned a university diploma.

**Table 3:** Magnitude of the effect of the implementation of the QPIP. Ratios of the after to before conditional total fertility rate by educational levels and provinces from Table 2

	Equation 3		Equation 4		Equation 4s	
	Quebec	Ontario	Quebec	Ontario	Quebec	Ontario
LTS	1.21	0.99	1.16	0.99	1.17	0.99
Secondary	1.21	0.98	1.42	0.90	1.46	0.91
NUPS	1.21	0.98	1.09	0.98	1.08	1.00
University	1.21	0.98	1.16	0.94	1.27	0.95

*LTS* Less than secondary, *NUPS* Non-university postsecondary education

### Sensitivity Analysis

We check the robustness of our results by estimating Eqs. 2 and 3 using linear probability rather than Poisson regression by comparing pairs of short periods and by comparing Quebec ‘before’ and ‘after’ periods, and by comparing Quebec to a group of selected other Canadian provinces.

The results from the estimation done using linear probability are reported in Table 7 in the Appendix with the detailed results from the estimation using control variables and sampling weights. The DiD coefficient is significant in all equations.

We define four short periods, two before the implementation of the QPIP—1996 to 2000 and 2001 to 2005—and two after—2007 to 2011 and 2012 to 2017. We compare them two by two by estimating Eq. 3 using sampling weights with control variables and using propensity weights. The detailed results are reported in Table 8 in the Appendix. Across the eight estimations, the DiD coefficient varies between 1.190 and 1.356 and is significant in all of them but one.

We compare Quebec to all other Canadian provinces but Ontario and Alberta. Alberta is known to have its own peculiar fertility dynamics distinct from that of Quebec but also from that of the other provinces. See Beaujot et al. (2013) and Mathieu et al. (2020). Comparing it to Quebec would be a special analysis and, given the differences between the fertility dynamics of the two provinces, it would not be a test of the implementation of the QPIP. I compare Quebec and the other provinces by estimating Eq. 3 using sampling weights with control variables and using propensity weights. The detailed results are reported in Table 9 in the Appendix. The DiD coefficient is of the same magnitude using the two estimations and is significant in the one done using sampling weights and control variables.

## Discussion

The implementation of the QPIP is clearly associated with the increase in fertility in Quebec. Estimating the fertility schedule within educational levels within each of the four groups defined according to the difference in differences method provides a nuanced insight of the effects of the new plan. Indeed, the effect varies according to educational levels. Women with university education, which had the highest conditional TFR before the implementation of the QPIP, still rank highest afterwards, but the increase is the highest among women who completed secondary education. The magnitude of the increase is notable: 17% for women who did not complete secondary education, 46% for those who completed it, 8% for those with non-university postsecondary education, and 27% for women who earned a university diploma. These figures may look large, but they are not surprising as the average TFR increased by 11.5% from the first to the second period we are comparing and the subpopulation we use in our analysis excludes unpartnered women whose fertility is comparatively low.

In theory, the high value of the conditional TFRs of university-educated women might be a consequence of discarding births occurred to women younger than 20 as such births are likely to be more common among the less educated whose fertility might otherwise rank higher. That said, the fertility of women aged less than 20 is low in Quebec. The sum of the ASFRs for Quebec women younger than 20, including the rare births occurring at age 12 or 13, decreased from 0.036 to 0.024 between 2014 and 2021 (Institut de la statistique du Quebec, 2022c). Considering births occurring before age 20 might increase slightly the conditional TFRs of low-educated women but given the magnitude of the sum of the corresponding rates, it is hard to see how it would change what can be learned from our results. Furthermore, fostering early births is not among the objectives of the Quebec family policy or of the QPIP, and including them in an assessment of the effect of the QPIP would be odd.

More likely, the comparatively high level of the fertility of university-educated women before the implementation of the QPIP is associated with their income, and quite certainly the magnitude of the increase in their fertility after its implementation is a consequence of the relatively generous benefits paid by the QPIP. Women who earn more are more likely to be able to afford to have children, but they may be deterred from having them, or some of them, because of the loss of earnings in the months that follow the birth and the consequences that leaving their job might have on their career. The protection given by labor law and the comparatively high replacement rate of the QPIP and maximum of insurable earnings seem to combine in a way that makes the effect of the plan greater for highly educated women. Thus, the QPIP meets the goal of helping women who do well on the labor market to have the children they want to have that was set in the 1997 statement on family policy. However, the large increase in the fertility of women who have completed secondary education and the small one in the fertility of women who have non-university postsecondary education do not have a straightforward explanation. These increases warrant further research.

Our extension of the differences in difference method to the modeling of the fertility schedule allowed us to test the effect of a family policy measure on fertility. Our use of integration to compute conditional TFRs from the modeled fertility schedules allowed to quantify the effect of the policy measure for several distinct categories of people—here women according to their educational level –and show that the effect of the policy varied across these categories.

Our results raise new questions as much as they answer some. The QPIP has increased the fertility of Quebec women, especially compared to that of Ontario women, but fertility is declining in both provinces since 2008. This decline is not a unique phenomenon: fertility is declining since 2008 in many countries and especially in countries known for the comprehensiveness of their family policy (Karlsdóttir et al., 2020; Hellstrand et al., 2021). What is intriguing in the case we study is the parallelism of the decline in the two provinces, in which the comparative gain induced by the QPIP is maintained while fertility decreases. This is an interesting riddle. It is not clear whether the method we introduce here can be further adapted to provide an answer to it. What is certain though is that while the data from the LFS allows comparing relatively large periods, it will not be enough to study the fine variation over time of the effect of a policy measure such as the QPIP. Statistics Canada has discontinued the household panel surveys it has initiated in the 1990s. Canada does not have registry data comparable to that of, say, Sweden (Ohlsson-Wijk & Andersson, 2022). Canadian demographers are hoping that administrative data and combination of administrative data from different sources might provide the information they need and do not have otherwise (Margolis et al., 2019a, 2019b). Whether or not this will prove fruitful is still to be seen.

## Figures and Tables

**Table 4:**
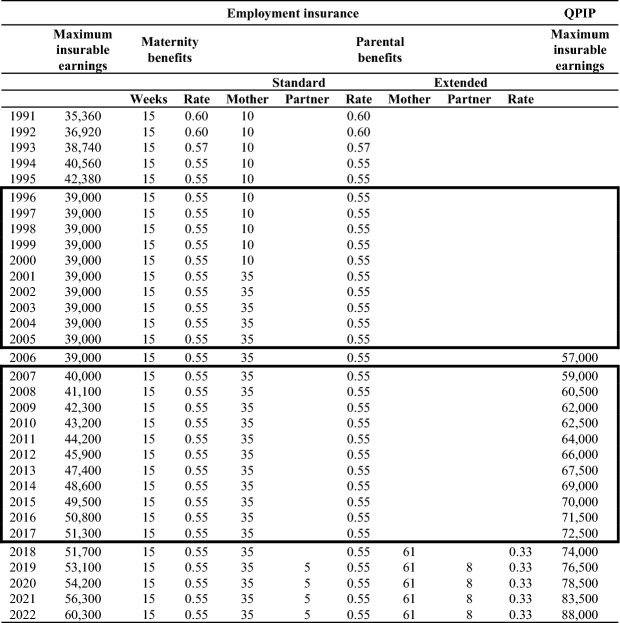
Parameters of the maternity and parental benefits from the Canadian unemployment insurance, 1991–2022. Maximum insurable earnings of the QPIP, 2006–2022. The two periods of the DiD design are marked using thick borders

Source: Human Resources Development Canada, (1996), Lin (1998); Statistics Canada (n. d.)

Maximum insurable earnings are in Canadian dollars (CAD). The maximum insurable earnings of the EI program were reduced to 39,000 CAD in 1996 and remained the same until 2007 when they began to be indexed on the average weekly earnings of the Industrial Aggregate in Canada as published by Statistics Canada (Employment Insurance Act S.C. 1996, c. 23, s. 4). The maximum insurable earnings of the QPIP are the maximum insurable earnings used by the Commission des normes, de l’équité, de la santé et de la sécurité du travail (Workplace Safety and Insurance Board) (Act respecting parental insurance, RLRQ c. A-29.011, s. 5). This amount was set to 33,000 CAD in 1985 and has since been indexed on the average weekly earnings of the Industrial Aggregate in Québec as established by Statistics Canada (Act respecting industrial accidents and occupational diseases, RLRQ c. A-3.001, s. 66)

## Data Availability

Our analyses use the confidential detailed microdata files of the Labor Force Survey available to researchers within secure facilities managed by Statistics Canada.

## Appendix

See Figs. 5, 6, 7, 8 and Tables 4, 5, 6, 7, 8, 9.

**Table 5:** Overview of the Quebec Parental Insurance Plan provisions for two-parent families

Type of Benefits	Basic Plan	Special Plan
Maternity	18 weeks	5 weeks
Non-shareable benefits	70% of earnings	75% of earnings
Paternity	5 weeks	3 weeks
Non-shareable benefits	70% of earnings	75% of earnings
Parental	32 weeks	25 weeks
Shareable benefits	First 7 weeks: 70% of earnings	75% of earnings
	Next 25 weeks: 55% of earnings	
	4 additional benefit weeks at 55% of earnings once 8 shareable parental benefit weeks have been paid to each parent	3 additional benefit weeks at 75% of earnings once 6 shareable parental benefit weeks have been paid to each parent

*Source*: Ministère du Travail, de l’Emploi et de la Solidarité sociale (2022)

**Table 6:** Some characteristics of the woman and her partner. Educational level of the woman and of the man (Proportions). Number of children of the woman in each age groups, age and income of the woman, age and income of the man (Means). Weekly employment income from the main job in constant 2020 Canadian dollars. Statistics are weighted using sampling weights or propensity weights

	Sampling weights				Propensity weights			
	Québec		Ontario		Québec		Ontario	
	Before	After	Before	After	Before	After	Before	After
Educational level of the woman								
< secondary	0.102	0.049	0.085	0.034	0.114	0.114	0.102	0.105
Secondary	0.179	0.104	0.227	0.152	0.178	0.180	0.185	0.189
NUPS	0.441	0.457	0.416	0.397	0.467	0.468	0.472	0.463
University	0.278	0.390	0.273	0.416	0.242	0.239	0.242	0.243
Educational level of the man								
< secondary	0.146	0.089	0.114	0.057	0.163	0.162	0.154	0.155
Secondary	0.169	0.131	0.211	0.185	0.168	0.167	0.174	0.178
NUPS	0.431	0.480	0.409	0.409	0.452	0.460	0.459	0.453
University	0.255	0.300	0.266	0.348	0.217	0.211	0.213	0.214
Number of children in each age group								
Aged 1 or 2	0.112	0.147	0.117	0.132	0.113	0.114	0.112	0.110
Aged 3 to 5	0.164	0.202	0.177	0.198	0.168	0.160	0.171	0.160
Aged 6 to 12	0.399	0.401	0.448	0.455	0.419	0.388	0.435	0.400
Aged 13 to 15	0.165	0.163	0.188	0.182	0.181	0.166	0.184	0.175
Aged 16 or 17	0.106	0.095	0.115	0.110	0.117	0.113	0.116	0.115
Aged 18 to 24	0.175	0.159	0.225	0.200	0.186	0.180	0.177	0.179
Average age and income of the woman								
Age	36.0	36.0	36.7	37.2	36.2	36.2	36.4	36.3
Income	806	978	908	1109	772	806	785	780
Average Age and income of the man								
Age	38.4	38.7	39.0	39.6	38.6	38.6	38.7	38.6
Income	1138	1265	1301	1445	1117	1136	1152	1131

**Table 7:** Detailed results of the estimation of Eqs. 2 and 3 using sampling weights and control variables. Detailed results of the estimation of Equations like Eqs. 2 and 3 but using linear probability, sampling weights and control variables

	Estimation with sampling weights and control variables		Estimation using linear probability, control variables and sampling weights	
	Equation 2	Equation 3	Like Eq. 2	Like Eq. 3
${e}^{{\beta }_{YT}}$ or *β*_*YT*_	1.256**	1.250**	0.006**	0.006**
Quebec [Ontario]	0.873*	0.878*	− 0.004**	− 0.004**
After [Before]	0.957	0.948	0.000	0.000
Woman’s age	2.238***		0.006***	
(Woman’s age)^2^	0.987***		0.000*	
Woman’s educational level				
Less than secondary		0.002*		0.077*
Secondary	0.849	0.000*	− 0.002	0.070**
NUPS	0.934	0.000*	0.000	− 0.025
University	0.990	0.000*	0.004*	− 0.158*
Woman’s age, Less than secondary		1.426**		0.077*
Woman’s age, Secondary		1.814*		0.070**
Woman’s age, PSNU		2.407*		− 0.025
Woman’s age, University		3.679*		0.000
(Woman’s age)^2^, Less than secondary		0.993*		0.000
(Woman’s age)^2^, Secondary		0.989*		0.000
(Woman’s age)^2^, PSNU		0.985*		0.000***
(Woman’s age)^2^, University		0.980*		0.000***
Woman’s income	1.008	1.004	0.000**	0.000*
Man’s income	1.011**	1.010**	0.000*	0.000*
Man’s age	0.988*	0.988**	0.000*	0.000*
Man’s educational level				
Secondary	1.053	1.067	0.001	0.001
NUPS	1.058	1.073	0.002	0.002
University	1.129	1.138	0.005**	0.005**
N children aged 1 or 2	1.217*	1.155*	0.013*	0.012^*^
N children aged 3 to 5	0.880	0.842*	− 0.005*	− 0.005*
N children aged 6 to 12	0.532*	0.537*	− 0.012*	− 0.012*
N children aged 13 to 15	0.563*	0.617*	− 0.007*	− 0.006*
N children aged 16 or 17	0.410*	0.463*	− 0.006*	− 0.005*
N children aged 18 to 24	0.691*	0.790	− 0.001**	− 0.001*
Intercept	0.000*		− 0.033**	

**p* < 0.05; ***p* < 0.01; **p* < 0.001

**Table 8:** Detailed results of the estimation of Eq. 3 using propensity weights and using sampling weights and control variables for comparing pairs of short periods before and after the implementation of the Quebec Parental Insurance Program. I: 1996–2000 and 2007–2011; II: 1996–2000 and 2002–2017; III: 2001–2005 and 23007–2011; IV: 2001–2005 and 2012–2017

	Estimation with propensity weights				Estimation with sampling weights and control variables			
	I	II	III	IV	I	II	III	IV
${e}^{{\beta }_{YT}}$	1.225*	1.250*	1.203*	1.232*	1.313*	1.356**	1.190	1.225*
Quebec [Ontario]	0.844*	0.848*	0.857*	0.861*	0.828*	0.814*	1.105	1.117
After [Before]	1.073	0.975	1.022	0.921	1.055	0.905	1.036	0.895
Woman’s educational level								
Less than secondary	0.061	0.004*	0.012	0.000**	0.006*	0.002**	0.003*	0.001*
Secondary	0.000***	0.001***	0.000***	0.000***	0.000***	0.000***	0.000***	0.000***
NUPS	0.000***	0.000***	0.000***	0.000***	0.000***	0.000***	0.000***	0.000***
University	0.000***	0.000***	0.000***	0.000***	0.000***	0.000***	0.000***	0.000***
Woman’s age, LTS	1.15	1.351	1.306	1.688*	1.293	1.441*	1.385	1.582*
Woman’s age, Sec	1.707***	1.580***	1.955***	1.798***	1.781***	1.782***	1.831***	1.797***
Woman’s age, PSNU	2.066***	2.027***	2.898***	2.920***	2.100***	2.209***	2.497***	2.697***
Woman’s age, Univ	2.688***	3.144***	4.363***	5.193***	2.792***	3.774***	3.488***	4.638***
(Woman’s age)^2^, LTS	0.995*	0.993**	0.993*	0.989**	0.995*	0.993***	0.994*	0.991*
(Woman’s age)^2^, Sec	0.989***	0.990***	0.987***	0.988***	0.989***	0.990***	0.988***	0.989***
(Woman’s age)^2^, PSNU	0.987***	0.987***	0.981***	0.981***	0.987***	0.987***	0.984***	0.983***
(Woman’s age)^2^, Univ	0.984***	0.981***	0.976***	0.974***	0.984***	0.980***	0.981***	0.976***
Woman’s income					1.003	1.001	1.008	1.006
Man’s income					1.015**	1.002	1.017**	1.005
Man’s age					0.992	0.988	0.987	0.983**
Man’s educational level [Less than secondary]								
Secondary					1.119	0.935	1.195	0.997
NUPS					1.253*	1.022	1.113	0.913
University					1.298*	1.180	1.055	0.947
N children aged 1 or 2					1.215***	1.233***	1.044	1.060
N children aged 3 to 5					0.798***	0.835**	0.825***	0.852**
N children aged 6 to 12					0.514***	0.595***	0.470***	0.548***
N children aged 13 to 15					0.710*	0.630**	0.597**	0.488***
N children aged 16 or 17					0.399***	0.529*	0.386**	0.527*
N children aged 18 to 24					0.732	0.898	0.634	0.815
Unmarried cohabitation [Marriage]							0.662***	0.614***

**p* < 0.05; ***p* < 0.01; **p* < 0.001

**Table 9:** Detailed results of the estimation of Eq. 3 using propensity weights and using sampling weights and control variables for comparing Quebec to all other Canadian provinces but Ontario and Alberta

	Estimation with propensity weights	Estimation with sampling weights and control variables
${e}^{{\beta }_{YT}}$	1.123	1.188*
Quebec [Other provinces]	0.985	0.958
After [Before]	1.082*	0.996
Woman’s educational level		
Less than secondary	0.003**	0.000***
Secondary	0.000***	0.000***
NUPS	0.000***	0.000***
University	1.404**	0.000***
Woman’s age, LTS	1.773***	1.724***
Woman’s age, Sec	2.311***	1.894***
Woman’s age, PSNU	3.009***	2.296***
Woman’s age, Univ	1.404**	3.237***
(Woman’s age)^2^, LTS	0.992***	0.990***
(Woman’s age)^2^, Sec	0.989***	0.989***
(Woman’s age)^2^, PSNU	0.985***	0.986***
(Woman’s age)^2^, Univ	0.982***	0.982***
Woman’s income		1.005
Man’s income		1.005
Man’s age		0.983***
Man’s educational level [Less than secondary]		
Secondary		1.206*
NUPS		1.208*
University		1.304**
N children aged 1 or 2		1.238***
N children aged 3 to 5		0.876**
N children aged 6 to 12		0.582***
N children aged 13 to 15		0.571***
N children aged 16 or 17		0.559**
N children aged 18 to 24		0.654**

**p* < 0.05; ***p* < 0.01; **p* < 0.001
